# Supplementary material for: Vitamin D and COVID-19 susceptibility and severity in the COVID-19 Host Genetics Initiative: A Mendelian randomization study
Source: PLoS Med. 2021 Jun 1;18(6):e1003605. doi: 10.1371/journal.pmed.1003605 (PMC8168855; doi:10.1371/journal.pmed.1003605)

**S3 Fig:** vitamin D synthesis pathway. For our sensitivity analyses, we restricted our instruments to variants in the circled genes below.


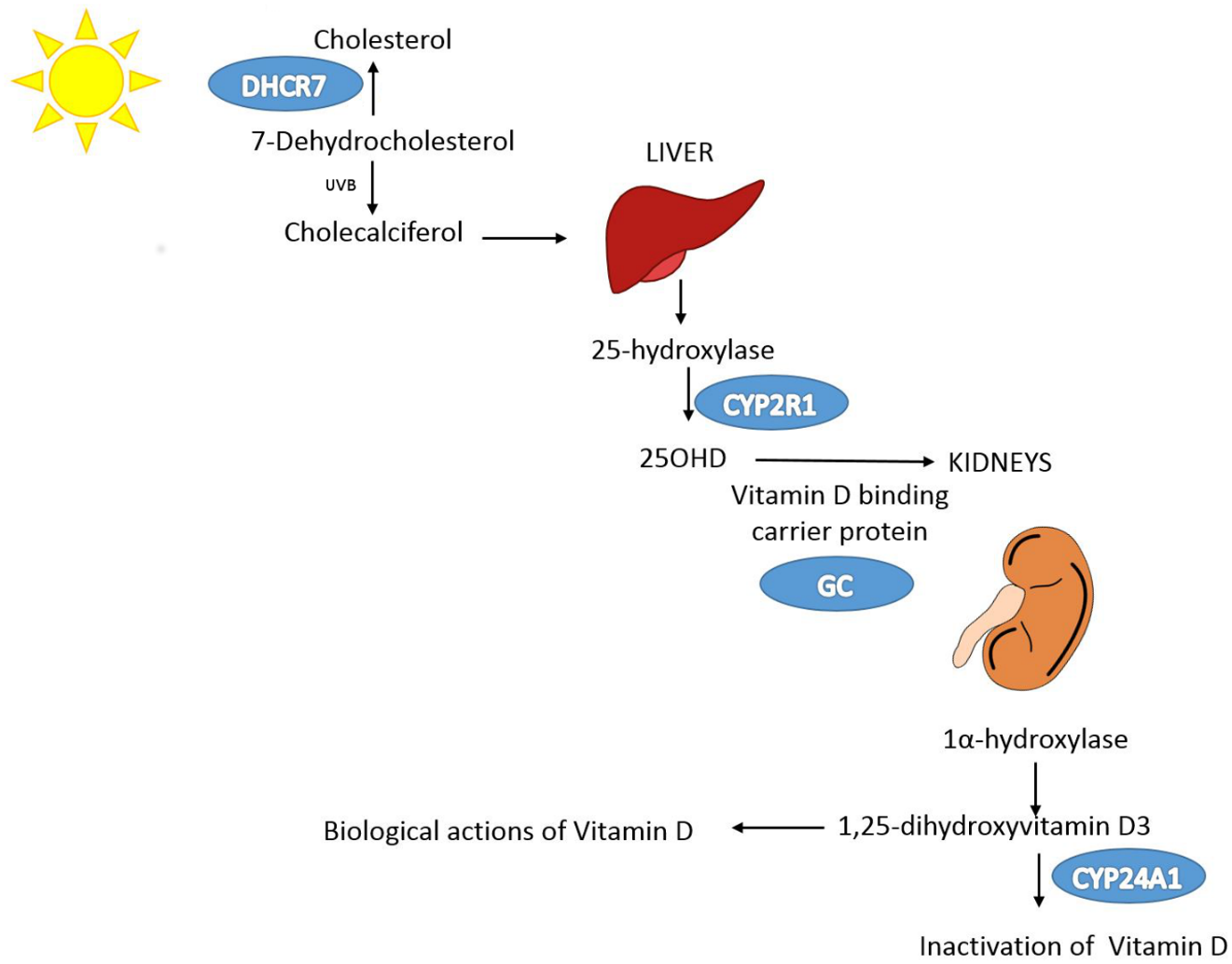

Supplement: S1 Fig — (DOCX) [file pmed.1003605.s004.docx]
