# Supplementary material for: Vitamin D and COVID-19 susceptibility and severity in the COVID-19 Host Genetics Initiative: A Mendelian randomization study
Source: PLoS Med. 2021 Jun 1;18(6):e1003605. doi: 10.1371/journal.pmed.1003605 (PMC8168855; doi:10.1371/journal.pmed.1003605)
Supplement: S1 Table — (DOCX) [file pmed.1003605.s005.docx]

**S5 Table**: Mendelian randomization sensitivity analyses results.

Odds ratios are given as the increase in odds of the Covid-19 outcome for every increase of one standard deviation log(25OHD). That is, an odds ratio greater than one indicates higher odds of the Covid-19 outcome. Confidence intervals are given using a Normal approximation, explaining minor discrepancies with p-values in cases close to the alpha=5% threshold of statistical significance.

| **Outcomes** | **Bootstrap MR Egger** | | **MR Egger** | | **Penalized Weighted Median** | | **Simple Mode** | | **Weighted Median** | | **Weighted Mode** | |
| --- | --- | --- | --- | --- | --- | --- | --- | --- | --- | --- | --- | --- |
|  | **OR (95% CI)** | **P** | **OR (95% CI)** | **P** | **OR (95% CI)** | **P** | **OR (95% CI)** | **P** | **OR (95% CI)** | **P** | **OR (95% CI)** | **P** |
| ***25OHD primary analysis with all SNPs*** | | | | | | | | | | | | |
| Susceptibility | 0.997 (0.85, 1.17) | 0.493 | 0.906 (0.766, 1.07) | 0.254 | 0.961 (0.819, 1.13) | 0.629 | 0.839 (0.56, 1.26) | 0.397 | 0.959 (0.817, 1.13) | 0.607 | 0.977 (0.849, 1.12) | 0.741 |
| Hospitalization | 1.15 (0.907, 1.47) | 0.122 | 1.08 (0.83, 1.4) | 0.568 | 1.04 (0.798, 1.34) | 0.792 | 0.887 (0.411, 1.91) | 0.761 | 1.03 (0.796, 1.34) | 0.811 | 1.12 (0.898, 1.41) | 0.31 |
| Severe Disease | 0.953 (0.702, 1.29) | 0.386 | 0.843 (0.623, 1.14) | 0.271 | 0.914 (0.672, 1.24) | 0.57 | 1.85 (0.835, 4.1) | 0.134 | 0.906 (0.668, 1.23) | 0.524 | 0.972 (0.751, 1.26) | 0.83 |
| ***25OHD sensitivity analysis restricted to genes in the vitamin D pathway*** | | | | | | | | | | | | |
| Susceptibility | 1.02 (0.861, 1.2) | 0.434 | 0.92 (0.717, 1.18) | 0.531 | 0.967 (0.832, 1.12) | 0.659 | 1.11 (0.819, 1.49) | 0.523 | 0.963 (0.826, 1.12) | 0.628 | 0.993 (0.84, 1.17) | 0.933 |
| Hospitalization | 1.07 (0.81, 1.4) | 0.315 | 0.849 (0.483, 1.49) | 0.583 | 1.05 (0.817, 1.34) | 0.716 | 0.918 (0.506, 1.67) | 0.785 | 1.04 (0.804, 1.34) | 0.769 | 1.09 (0.866, 1.39) | 0.467 |
| Severe Disease | 0.883 (0.642, 1.22) | 0.236 | 0.666 (0.414, 1.07) | 0.127 | 0.897 (0.663, 1.22) | 0.483 | 1.28 (0.651, 2.52) | 0.491 | 0.89 (0.657, 1.21) | 0.453 | 0.87 (0.633, 1.2) | 0.412 |
| ***25OHD sensitivity analysis after removal of SNPs identified by Phenoscanner*** | | | | | | | | | | | | |
| Susceptibility | 1.36 (0.954, 1.94) | 0.04 | 0.896 (0.578, 1.39) | 0.637 | 1.16 (0.899, 1.49) | 0.258 | 0.686 (0.434, 1.08) | 0.145 | 1.09 (0.826, 1.43) | 0.557 | 1.11 (0.831, 1.49) | 0.491 |
| Hospitalization | 1.73 (0.957, 3.14) | 0.031 | 0.91 (0.359, 2.31) | 0.848 | 1.5 (0.957, 2.35) | 0.0769 | 0.442 (0.2, 0.977) | 0.0785 | 1.33 (0.843, 2.09) | 0.221 | 1.43 (0.909, 2.24) | 0.161 |
| Severe Disease | 1.88 (0.906, 3.9) | 0.044 | 0.767 (0.329, 1.79) | 0.56 | 1.3 (0.768, 2.19) | 0.329 | 1.4 (0.61, 3.21) | 0.45 | 1.27 (0.729, 2.21) | 0.399 | 1.24 (0.682, 2.24) | 0.504 |
