# Supplementary material for: Vitamin D and COVID-19 susceptibility and severity in the COVID-19 Host Genetics Initiative: A Mendelian randomization study
Source: PLoS Med. 2021 Jun 1;18(6):e1003605. doi: 10.1371/journal.pmed.1003605 (PMC8168855; doi:10.1371/journal.pmed.1003605)
Supplement: S2 Text — (DOCX) [file pmed.1003605.s007.docx]

**S7 Text:** GEN-COVID Multicenter Study **(**[**https://sites.google.com/dbm.unisi.it/gen-covid**](https://sites.google.com/dbm.unisi.it/gen-covid)**)**

Francesca Montagnani^3,5^, Chiara Fallerini^1^, Margherita Baldassarri^1^, Annarita Giliberti^1^, Elisa Benetti^3^, Floriana Valentino^1^, Sergio Daga^1^, Gabriella Doddato^1^, Susanna Croci^1^, Rossella Tita^2^, Francesca Fava^1,2^, Mirella Bruttini^1,2^, Elisa Frullanti^1^, Anna Maria Pinto^2^, Francesca Mari^1,2^, Simone Furini^3^, Laura Di Sarno^1^, Andrea Tommasi^1,2^, Maria Palmieri^1^, Arianna Emiliozzi^3,4^, Massimiliano Fabbiani^4^, Barbara Rossetti^4^, Giacomo Zanelli^3,4^, Laura Bergantini^5^, Miriana D’Alessandro^5^, Paolo Cameli^5^, David Bennet^5^, Federico Anedda^6^, Simona Marcantonio^6^, Sabino Scolletta^6^, Federico Franchi^6^, Maria Antonietta Mazzei^7^, Edoardo Conticini^8^, Luca Cantarini^8^, Bruno Frediani^8^, Danilo Tacconi^9^, Marco Feri^10^, Raffaele Scala^11^, Genni Spargi^12^, Marta Corridi^12^, Cesira Nencioni^13^, Gian Piero Caldarelli^14^, Maurizio Spagnesi^15^, Paolo Piacentini^15^ , Maria Bandini^15^, Elena Desanctis^15^, Anna Canaccini^16^, Chiara Spertilli^9^, Alice Donati^10^, Luca Guidelli^11^, Leonardo Croci^13^, Agnese Verzuri^16^, Valentina Anemoli^16^, Agostino Ognibene^17^, Massimo Vaghi^18^, Antonella D’Arminio Monforte^19^, Esther Merlini^19^, Mario U. Mondelli^20,21^, Stefania Mantovani^20^, Serena Ludovisi^20,21^, Massimo Girardis^22^, Sophie Venturelli^22^, Marco Sita^22^, Andrea Cossarizza^23^, Andrea Antinori^24^, Alessandra Vergori^24^, Stefano Rusconi^25,26^, Matteo Siano^26^, Arianna Gabrieli^26^, Agostino Riva^25,26^, Daniela Francisci^27,28^, Elisabetta Schiaroli^27^, Pier Giorgio Scotton^29^, Francesca Andretta^29^, Sandro Panese^30^, Renzo Scaggiante^31^, Saverio Giuseppe Parisi^32^, Francesco Castelli^33^, Maria Eugenia Quiros-Roldan^33^, Paola Magro^33^, Cristina Minardi^33^, Deborah Castelli^33^, Itala Polesini^33^, Matteo Della Monica^34^, Carmelo Piscopo^34^, Mario Capasso^35,36,37^, Roberta Russo^35,36^, Immacolata Andolfo^35,36^, Achille Iolascon^35,36^, Massimo Carella^38^, Marco Castori^38^, Giuseppe Merla^38^, Filippo Aucella^39^, Pamela Raggi^40^, Carmen Marciano^40^, Rita Perna^40^, Matteo Bassetti^41,42^, Antonio Di Biagio^42^, Maurizio Sanguinetti^43,44^, Luca Masucci^43,44^, Chiara Gabbi^45^, Serafina Valente^46^, Susanna Guerrini^8^, Ilaria Meloni^1^, Maria Antonietta Mencarelli^2^, Caterina Lo Rizzo^2^, Elena Bargagli^6^, Marco Mandalà^47^, Alessia Giorli^47^, Lorenzo Salerni^47^, Giuseppe Fiorentino^48^, Patrizia Zucchi^49^, Pierpaolo Parravicini^49^, Elisabetta Menatti^50^, Stefano Baratti^51^, Tullio Trotta^52^, Ferdinando Giannattasio^52^, Gabriella Coiro^52^, Fabio Lena^53^, Domenico A. Coviello^54^, Cristina Mussini^55^

1. Medical Genetics, University of Siena, Italy
2. Genetica Medica, Azienda Ospedaliera Universitaria Senese, Italy
3. Dept of Medical Biotechnologies, University of Siena, Italy
4. Dept of Specialized and Internal Medicine, Tropical and Infectious Diseases Unit
5. Unit of Respiratory Diseases and Lung Transplantation, Department of Internal and Specialist Medicine, University of Siena
6. Dept of Emergency and Urgency, Medicine, Surgery and Neurosciences, Unit of Intensive Care Medicine, Siena University Hospital, Italy
7. Department of Medical, Surgical and Neuro Sciences and Radiological Sciences, Unit of Diagnostic Imaging, University
8. Rheumatology Unit, Department of Medicine, Surgery and Neurosciences, University of Siena, Policlinico Le Scotte, Italy
9. Department of Specialized and Internal Medicine, Infectious Diseases Unit, San Donato Hospital Arezzo, Italy
10. Dept of Emergency, Anesthesia Unit, San Donato Hospital, Arezzo, Italy
11. Department of Specialized and Internal Medicine, Pneumology Unit and UTIP, San Donato Hospital, Arezzo, Italy
12. Department of Emergency, Anesthesia Unit, Misericordia Hospital, Grosseto, Italy
13. Department of Specialized and Internal Medicine, Infectious Diseases Unit, Misericordia Hospital, Grosseto, Italy
14. Clinical Chemical Analysis Laboratory, Misericordia Hospital, Grosseto, Italy
15. Department of Prevention, Azienda USL Toscana Sud Est, Italy
16. Territorial Scientific Technician Department, Azienda USL Toscana Sud Est, Italy
17. Clinical Chemical Analysis Laboratory, San Donato Hospital, Arezzo, Italy
18. Chirurgia Vascolare, Ospedale Maggiore di Crema, Italy
19. Department of Health Sciences, Clinic of Infectious Diseases, ASST Santi Paolo e Carlo, University of Milan, Italy
20. Division of Infectious Diseases and Immunology, Fondazione IRCCS Policlinico San Matteo, Pavia, Italy
21. Department of Internal Medicine and Therapeutics, University of Pavia, Italy
22. Department of Anesthesia and Intensive Care, University of Modena and Reggio Emilia, Modena, Italy
23. Department of Medical and Surgical Sciences for Children and Adults, University of Modena and Reggio Emilia,

Modena, Italy

1. HIV/AIDS Department, National Institute for Infectious Diseases, IRCCS, Lazzaro Spallanzani, Rome, Italy
2. III Infectious Diseases Unit, ASST-FBF-Sacco, Milan, Italy
3. Department of Biomedical and Clinical Sciences Luigi Sacco, University of Milan, Milan, Italy
4. Infectious Diseases Clinic, Department of Medicine 2, Azienda Ospedaliera di Perugia and University of Perugia,

Santa Maria Hospital, Perugia, Italy

1. Infectious Diseases Clinic, "Santa Maria" Hospital, University of Perugia, Perugia, Italy
2. Department of Infectious Diseases, Treviso Hospital, Local Health Unit 2 Marca Trevigiana, Treviso, Italy
3. Infectious Diseases Department, Ospedale Civile "SS. Giovanni e Paolo", Venice, Italy
4. Infectious Diseases Clinic, ULSS1, Belluno, Italy
5. Department of Molecular Medicine, University of Padova, Italy
6. Department of Infectious and Tropical Diseases, University of Brescia and ASST Spedali Civili Hospital, Brescia, Italy
7. Medical Genetics and Laboratory of Medical Genetics Unit, A.O.R.N. "Antonio Cardarelli", Naples, Italy
8. Department of Molecular Medicine and Medical Biotechnology, University of Naples Federico II, Naples, Italy
9. CEINGE Biotecnologie Avanzate, Naples, Italy
10. IRCCS SDN, Naples, Italy
11. Division of Medical Genetics, Fondazione IRCCS Casa Sollievo della Sofferenza Hospital, San Giovanni Rotondo,

Italy

1. Department of Medical Sciences, Fondazione IRCCS Casa Sollievo della Sofferenza Hospital, San Giovanni

Rotondo, Italy

1. Clinical Trial Office, Fondazione IRCCS Casa Sollievo della Sofferenza Hospital, San Giovanni Rotondo, Italy
2. Department of Health Sciences, University of Genova, Genova, Italy
3. Infectious Diseases Clinic, Policlinico San Martino Hospital, IRCCS for Cancer Research Genova, Italy
4. Microbiology, Fondazione Policlinico Universitario Agostino Gemelli IRCCS, Catholic University of Medicine,

Rome, Italy

1. Department of Laboratory Sciences and Infectious Diseases, Fondazione Policlinico Universitario A. Gemelli IRCCS,

Rome, Italy

1. Independent Scientist, Milan, Italy
2. Department of Cardiovascular Diseases, University of Siena, Siena, Italy
3. Otolaryngology Unit, University of Siena, Italy
4. AORN dei Colli Presidio Ospedaliero Cotugno, Italy
5. Department of Internal Medicine, ASST Valtellina e Alto Lario, Sondrio, Italy
6. Study Coordinator Oncologia Medica e Ufficio Flussi Sondrio, Italy
7. Department of Infectious and Tropical Diseases, University of Padova, Padova, Italy
8. First Aid Department, Luigi Curto Hospital, Polla, Salerno, Italy
9. Local Health Unit-Pharmaceutical Department of Grosseto, Toscana Sud Est Local Health Unit, Grosseto, Italy
10. U.O.C. Laboratorio di Genetica Umana, IRCCS Istituto G. Gaslini, Genoa, Italy.
11. Infectious Diseases Clinics, University of Modena and Reggio Emilia, Modena, Italy.
