# Supplementary material for: Vitamin D and COVID-19 susceptibility and severity in the COVID-19 Host Genetics Initiative: A Mendelian randomization study
Source: PLoS Med. 2021 Jun 1;18(6):e1003605. doi: 10.1371/journal.pmed.1003605 (PMC8168855; doi:10.1371/journal.pmed.1003605)
Supplement: Attachment — Submitted filename: Rebuttal VitD PlosMed v2.docx [file pmed.1003605.s008.docx]

Requests from the editors:

1.Abstract: Please structure your abstract using the PLOS Medicine headings (Background, Methods and Findings, Conclusions). Please combine the Methods and Results sections into one section, “Methods and findings”.

**This was done as suggested.**

2. Abstract: Methods: Please provide some background demographic information on the participants contributing to the GWAS used to obtain variants associated with 25OHD, GWAS for Covid-19.

**We have modified the last sentence of the abstract method section as follows:**

***“Cohorts from the Covid-19 Host Genetics Initiative GWAS included up to 14,134 individuals with Covid-19, and 1,284,876 with Covid-19, from 11 countries. Analyses were restricted to individuals of European descent when possible.”***

**Note that due to confidentiality and legal concerns which would have limited the Covid-19 HGI effort, full patient and cohort level data is not available through the Covid-19 HGI initiative. However, each cohort followed the same analysis and genotype quality control framework, which we have now described in the main text method section (see also Reviewer 2’s second point).**

3. Abstract: Line 85: Please provide the p values associated with the OR along with the 95% CIs.

**This was done as suggested.**

4. Abstract: Line 86-87: This sentence could be clarified or expanded upon, as it seemed from the results that there was some variation in the findings between the primary and secondary/sensitivity analyses.

**This does not apply anymore. The observed effects with the updated and enlarged Covid-19 Host Genetics Initiative meta-analysis summary statistics are now consistently null.**

5. Abstract: In the last sentence of the Abstract Methods and Findings section, please describe the main limitation(s) of the study's methodology.

**We have added the following:**

***“******These results may be limited by weak instrument bias in some analyses. Further, our results do not apply to individuals with vitamin D deficiency.”***

6. Abstract: Conclusions: Please address the study implications without overreaching what can be concluded from the data; the phrase "In this study, we observed ..." may be useful. At lines 91-92, we suggest softening the language/recommendation here-“...individuals should not use vitamin D supplements…” to focus instead on what the data do and do not support (e.g. “...these findings do not provide supporting evidence for the use of vitamin D…”)

**We have changed the conclusion section to the following:**

***“Our results do not support that patients be advised to take vitamin D supplementation to protect against Covid-19 outcomes. Further, other therapeutic or preventative avenues should be given higher priority for Covid-19 randomized controlled trials.”***

7. Author Summary: At this stage, we ask that you include a short, non-technical Author Summary of your research to make findings accessible to a wide audience that includes both scientists and non-scientists. The Author Summary should immediately follow the Abstract in your revised manuscript. This text is subject to editorial change and should be distinct from the scientific abstract. Please see our author guidelines for more information: <https://journals.plos.org/plosmedicine/s/revising-your-manuscript#loc-author-summary>

**This was done as instructed.**

8. Throughout text: Please use square brackets for in-text citations, like this [1].

**We have now used Mendeley’s *PLOS Medicine* reference style. This has changed the citations to square brackets as specified.**

9. Methods: Prospective protocol: Did your study have a prospective protocol or analysis plan? Please state this (either way) early in the Methods section.

**Please see our answer following part c).**

a) If a prospective analysis plan (from your funding proposal, IRB or other ethics committee submission, study protocol, or other planning document written before analyzing the data) was used in designing the study, please include the relevant prospectively written document with your revised manuscript as a Supporting Information file to be published alongside your study, and cite it in the Methods section. A legend for this file should be included at the end of your manuscript.

**Please see our answer following part c).**

b) If no such document exists, please make sure that the Methods section transparently describes when analyses were planned, and when/why any data-driven changes to analyses took place.

**Please see our answer following part c).**

c) In either case, changes in the analysis-- including those made in response to peer review comments-- should be identified as such in the Methods section of the paper, with rationale.

**We added the following after first paragraph of the method section, which will answer points 9abc above:**

***“Our study did not employ a prospective protocol. Analyses were first planned and performed in July 2020 and updated following peer-review in December 2020. Three major changes were made during the update. First, we used the most up to date Covid-19 Host Genetics Initiative (Covid-19 HGI) genome-wide association study summary statistics. These were made available during the peer-review process. Second, to alleviate potential selection and collider bias, we modified the outcome phenotypes to include population controls. Lastly, we performed additional mendelian randomization sensitivity analyses to check for the robustness of our results. The latter two modifications were made at the request of peer-reviewers.”***

10. Results: Lines 276-281: Please clarify for the MR Egger estimates of 25OHD and hospitalization that effects did not reach statistical significance, in contrast to what you saw with the primary IVW analysis for hospitalization.

**The observed effects with the updated Covid-19 Host Genetics Initiative meta-analysis summary statistics are now consistently null. Therefore, they are no longer discordant.**

11. Results: Line 273, 287: In these two instances, the term "trend" is used- once where statistical significance is not reached, and once where it is reached. The term “trend” should be used only when the test for trend has been conducted. Please revise accordingly.

**The observed effects with the updated Covid-19 Host Genetics Initiative meta-analysis summary statistics are now consistently null and the word “trend” has now been removed.**

12. Results: Line 315: Please clarify if this is the result for susceptibility with the extended phenotypes definition.

**We have clarified our references to each analysis throughout the text.**

13. Results: In presenting the results, it would be helpful to more explicitly point out consistencies and inconsistencies between analyses by clarifying where secondary analyses differed from the main results, although you do allude to this where you mention widening confidence intervals (for example, at line 300-303: “While confidence intervals widened, effect estimates when restricting our analysis to these 22 SNPs remained similar for susceptibility (0.77; 95% CI: 0.48, 1.23; P=0.27), hospitalization (2.89; 95% CI: 1.18, 7.06; P=0.02), and severe disease (2.52; 95% CI: 0.63, 10.0; P=0.19), though this did not reach statistical significance in the latter case.”

**The observed effects with the updated Covid-19 Host Genetics Initiative meta-analysis summary statistics are now consistently null.**

14. Discussion: Please slightly re-organize the Discussion as follows: a short, clear summary of the article's findings; what the study adds to existing research and where and why the results may differ from previous research; strengths and limitations of the study; implications and next steps for research, clinical practice, and/or public policy; one-paragraph conclusion.

**Thank you for this comment. The discussion was rewritten using this template.**

15. Figure 1: This diagram might be more appropriate as a supporting information file. Please define all abbreviations used in the figure in the Figure legend. It might be helpful to call attention to particular points of the pathway most relevant for your study (e.g. particular genes involved.

**This was done as suggested and moved to the new Supplement 2.**

16. Figure 2 and supporting information Figure 2.1 (in Supplement 3): Please provide the labels for the X axis.

**This figure was completely redesigned to add more mendelian randomization methods, at the request of the reviewers. Labels were made clearer as suggested. Supplement 3 was removed, at the request of professor Burgess (reviewer 3), who suggested to make it the primary analyses.**

17. Checklist: Please ensure that the study is reported according to the STROBE guideline, and include the completed STROBE checklist as Supporting Information. When completing the checklist, please use section and paragraph numbers, rather than page numbers. Please add the following statement, or similar, to the Methods: "This study is reported as per the Strengthening the Reporting of Observational Studies in Epidemiology (STROBE) guideline (S1 Checklist)."

**We have now provided the STROBE checklist. Note that we had previously used the STREGA checklist, which is the STROBE extension for genetic association studies. While neither STROBE nor STREGA completely apply to mendelian randomization studies, if editors prefer that we use STREGA, we can undertake this.**

18. Data availability statement: Thank you for noting that the SNPs are available in published studies. If it is possible, it would be helpful to have a summary supporting information file, or a more specific reference (DOI/accession number) to find the repository genetic instrument SNPs used in your study.

**The Covid-19 Host Genetics Initiative summary statistics are hosted directly on their website, in the results section, and do not have a DOI or accession number. We have updated the address so that it now leads directly to the results section and have specified which data freeze we have used for our study.**

Comments from the reviewers:

Reviewer #1: Overall comments: Thank you very much for the opportunity to review this manuscript entitled "Vitamin D and Covid-19 Susceptibility and Severity: a Mendelian Randomization Study". This is an interesting topic of research, which should be of importance to Covid-19. However, prior to publication, I feel that the manuscript needs some substantial work to evaluate the reliability of the results and explain to the readers how to interpret the results.

**We thank the reviewer for the positive review and the suggestions to improve on our manuscript.**

The authors used two-sample Mendelian randomization analysis to determine the role of vitamin D status in Covid-19. As the authors suggest, MR is a powerful method for inferring causality, which seems to replace RCTs. However, its careless use can lead to many false results, especially for two-sample Mendelian randomization study. The authors made a conclusion "genetically increased 25OHD levels did not protect against Covid-19 susceptibility, or severity, and in some analyses was associated with worsened outcomes, which support vitamin D supplementation to prevent Covid-19 outcomes". Actually, the essence of MR is used to explore the causal relationship of risk factors to the outcome, but it could not replace RCT to explore the effect of reducing risk factors on the outcome. Therefore, I don't think this conclusion is appropriate. How this finding can guide the impact of vitamin D supplements on COVID-19 needs to be further explored.

**We do not feel that Mendelian randomization should replace randomized controlled trials, but rather could be used to prioritize them. There is considerable literature on the use of genetic determinants of disease on drug development or repurposing (e.g. PMID: 32860016 and 32895551). In the case of Vitamin D, Mendelian randomization has consistently predicted the result of clinical trials on Vitamin D supplementation. In a pandemic, we think it would be wise to leverage this technique to better allocate our limited time and resources to trials with a higher “pre-test” probability of working.**

**The conclusion was changed as shown above in the response to editors’ comments, and we have further modified the following sentence in the introduction to clarify this point:**

***“MR has been used in conjunction with proteomics and metabolomics to prioritize drug development and repurposing, and support investment in RCTs which have a higher chances of success[17,18]. In the case of vitamin D, MR has been able to provide causal effect estimates consistently in line with those obtained from RCTs[10,19–23], and may support investments in 25OHD supplementation trials in Covid-19, if a benefit was shown.”***

Specific Comments:

Abstract:

1. I would reword "GWASs of Covid-19 susceptibility and severity from the Covid-19 Host Genetics Initiative were used to test the effect of 25OHD levels on these outcomes" It's not entirely clear what the authors mean. Do they mean that GWASs of Covid-19 susceptibility and severity from the Covid-19 Host Genetics Initiative were used to perform instrument-outcome associations?

**The reviewer understood this correctly, and we have clarified this sentence in the abstract as follows:**

***“GWASs of Covid-19 susceptibility and severity from the Covid-19 Host Genetics Initiative were used as outcomes.”***

2. The sentence "the genetically increased 25OHD levels tended to increase the odds ratio of severe disease, while OR = 2.21; 95% CI: 0.87-5.55" should be reworded. The finding is not consistent with its explanation.

**This does not apply anymore. The observed effects with the updated Covid-19 Host Genetics Initiative meta-analysis summary statistics are now consistently null.**

3. Needs for more detailed information of Extensive sensitivity analyses "Extensive sensitivity analyses probing the assumptions of MR provided consistent estimates.". In addition, the result seems to be inconsistent in different instrumental variables and different methods.

**With the updated results from the enlarged COVID-19 HGI GWAS meta-analyses, the analyses now consistently demonstrate a lack of effect. Recognizing the limited amount of space afforded to our explanation on the sensitivity analyses performed, we have modified the sentence as follows:**

***“We used an additional 6 meta-analytic methods, as well as sensitivity analysis after removal of variants at risk of horizontal pleiotropy, and obtained similar results.”***

Introduction

4. Needs some rewording: " In the case of vitamin D, MR has been able to provide causal effect estimates consistently in line with those obtained from RCTs, and would therefore support investments in 25OHD supplementation trials in Covid-19, if a benefit was shown". How to balance the findings of MR study and RCT?

**As mentioned in our response above, we do not believe there is a balance to be struck here, but rather there is a hierarchy and chronology of evidence to follow. That is, if a well-designed randomized controlled trial is performed, then its conclusions would likely be more relevant than MR. However, well-designed trials are difficult to perform, and in cases where a readily available trial prioritization tool exists (such as MR with vitamin D), we believe that the tool should be used. In a pandemic, choosing the right trials to prioritize becomes even more important. We have clarified this chronology of evidence in the introduction, as per our response to this reviewer’s first comment.**

5. Aside from the mentioned several core assumptions of MR, it is important to note the linearity assumptions within the IV framework.

**We have made the following change as suggested (and refer to this reviewer’s 13^th^ comment for more on the importance of this assumption in understanding our results):**

***“Specifically, MR also assumes that the relationship between the exposure and the outcome is linear. However, this assumption still provides valid results when studying population-level effects[25].”***

Method

6. The authors should provide heterogeneity tests of individual genetic variants to explore the robust of IVW method.

**This was done as suggested.**

7. More sensitivity analysis methods, including but not limited to the weighted median and penalized weighted median methods, should be carried out.

**This was done as suggested, and we thank the reviewer for this comment, which strengthened our results. Specifically, in addition to MR Egger, we also performed bootstrap MR Egger, weighted median, penalized weighted median, simple mode, and weighted mode MR. None of these methods provided convincing evidence of a vitamin D protective effect on Covid-19. The justification for each was given in the methods section as follows:**

***“We undertook extensive analysis to assess the risk of horizontal pleiotropy (a violation of the second MR assumption). First, we used MR Egger method, which allows for an additional intercept (alpha) term which also provides an estimate of directional horizontal pleiotropy. Given possible instability in MR Egger estimates[32], we also used the bootstrap MR Egger method to meta-analyze the causal effect estimates from each SNP instrument. Further, we used four additional meta-analysis methods known to be more robust to presence of horizontal pleiotropy (at the expense of statistical power): penalised weighted median, simple mode, weighted median, and weighted mode[33].”***

8. As far as I know, the COVID-19 host genetics initiative provided the summary data for severe COVID-19, COVID-19 and hospitalized COVID-19 with control groups were subjects from the general population without the specific phenotype, subjects who were COVID-19 negative based on prediction or self-report, or subjects who had COVID-19 without hospitalization. The author used extended phenotypes, but limited the controls as subjects who were COVID-19 negative based on prediction or self-report, or subjects who had COVID-19 without hospitalization. The authors should give a reason for the selected controls.

**The Covid-19 HGI provides summary statistics for both types of analyses (i.e. population controls, as well as covid-19 tested controls), which can be found in their results section (**[**https://www.covid19hg.org/results/**](https://www.covid19hg.org/results/)**). However, given astute observations from Professor Burgess (reviewer 3) on the risk of selection and collider bias when using only controls who were tested for Covid-19, we followed his advice to now only use population controls (analyses A2, B2, and C2 on the Covid-19 HGI website).**

9. The definition of exposure is unclear. The detailed information of exposure- vitamin D and the information of summary data for the selected SNP associated with vitamin D should be provided.

**We agree with the reviewer, and we have now provided the full list of SNPs and their relevant summary statistics in Supplement 3.**

Result

10. The description of the results seems to be inconsistent with the results in the table, such as, the MR Egger results are inconsistent with Egger OR as the Table 2 showed. In addition, the result seems to be inconsistent in different instrumental variables and different methods.

**This does not apply anymore. The observed effects with the updated Covid-19 Host Genetics Initiative meta-analysis summary statistics are now consistently null.**

Discussion

11. The increasing observational studies indicate that vitamin D deficiency is associated with increased COVID-19 risk[1, 2]. The authors should explain more deeply for the contradictory results in the discussion. In addition, the findings should be interpreted more cautiously for guiding future 25OHD supplementation trials.

**As mentioned in our introduction, vitamin D is highly correlated with other clear risk factors for worsened Covid-19 outcomes. In general, older patients with more comorbidities and more risk of institutionalization all tend to have lower vitamin D levels. Observational studies reporting on the association between vitamin D and most health outcomes are therefore generally unreliable, as can be seen from the lack of RCTs showing beneficial effect on almost all human diseases for which it has been tested (despite all being supported by observational evidence). We have therefore added the following to the conclusion:**

***“These findings highlight the confounded association between vitamin D and Covid-19 due to factors such as older age, institutionalization, or medical comorbidities, that are all linked to lower vitamin D levels and cannot be controlled for even when using advanced statistical adjustments. Therefore, in contrast to observational studies, our findings do not support vitamin D supplementation to prevent worse Covid-19 outcomes.”***

12. However, the unmeasured confounders or alternative causal pathways may be still affected those results because of the limitation of the method. A previous study found that serum 25OHD concentrations are highly heritable is winter season, which suggests a varied role of genetic factors, dependent on the UVB intensity[3]. So how to reduce the bias of UVB against results? I suggested that it should be added as a limitation.

**Given that genetic variants are assigned at birth before disease onset, MR controls for non-genetic confounders. In the case of the gene by environment effect in winter months suggested by the reviewer, the study they refer to suggests that while vitamin D levels are heritable in winter, this is not the case in summer, where environmental factors are more important. However, the meta-analysis of vitamin D genetic variants controlled for seasonal factors, and therefore provide average season-independent variants associated with vitamin D levels. While this would alleviate some of the bias, not accounting for season in our Covid-19 outcome GWAS may still lead to averaging the effect of vitamin D on Covid-19 over all months of the pandemic. We therefore recognize that some effect of vitamin D might be attenuated. However, we emphasize that it is impossible to determine if this effect would be beneficial or detrimental to Covid-19 outcome. We have added the following in the methods section:**

***“To find genetic variants explaining 25OHD levels[27], we used a GWAS from our group, which is the largest published GWAS of 25OHD levels. Importantly, this meta-analysis controlled for vitamin D levels season of vitamin D measurement to obtain genetic variants significantly associated with 25OHD levels.”***

**We have also acknowledged this limitation as follows:**

***“given that vitamin D levels are affected by season (with higher levels after sunlight exposure), even if our SNP-instruments were obtained from a GWAS that controlled for season of blood draw, there is still a chance for effect attenuation by averaging the effect of 25OHD levels on Covid-19 over all seasons. Nevertheless, the size of this bias is likely small, and its direction undetermined (i.e. it could be hiding a detrimental effect of vitamin D).”***

**Lastly, on the roles of alternative pathways, we have used the largest meta-analysis on the genetic determinants of 25OHD levels. This meta-analysis already found multiple independent genetic variants known to be in genes directly involved in vitamin D metabolism. Therefore, by using additional genetic variants in our MR, we would be exploring alternative genetic causal pathways and increasing statistical power, but we would also increase the risk of horizontal pleiotropy thereby risking the invalidation our results. We believe our analysis strikes a good balance between statistical power and risk of horizontal pleiotropy.**

13. As mentioned above, it is important to note the linearity assumptions within the IV framework. If the effect of vitamin D supplementation on Covid-19 may be nonlinear, how to interpret this finding?

**While we recognize that an additive effect may miss some of the variance in Covid-19 outcomes explained by vitamin D levels, the only biologically plausible non-linear situation where the estimated MR association would be falsely nullified is in the case of a U-shaped response. For example, if both very low and very high vitamin D levels conferred protective effect. There is no biological or observational support for such a situation. Therefore, while our effect estimates might be biased, the direction and the presence of a protective effect of vitamin D on Covid-19 is unlikely to be completely missed (unless it is very small, and assuming that there is an effect). This is further supported by simulation studies (e.g. PMID 25166881, from Burgess et al.) showing that in the presence of non-linear exposure-outcome relationships, using linear effect model still provides adequate estimates when using population-based methods. Given that we have now allowed for the use of population-based controls, we suspect that non-linearity is unlikely to cause significant bias.**

**We have added the following to our limitation paragraph to explain these issues:**

***“Lastly, our MR analyses assumes a linear relationship the exposure-outcome relationship. While this may slightly bias our results, simulation studies have previously shown that this assumption provides adequate results when looking at a population effect[25]. Therefore, for the purpose vitamin D supplementation RCT prioritization, our conclusions should still be valid.”***

Referrences

1) Meltzer Do Auid- Orcid: --- Fau - Best TJ, Best Tj Fau - Zhang H, Zhang H Fau - Vokes T, Vokes T Fau - Arora V, Arora V Fau - Solway J, Solway J. Association of Vitamin D Deficiency and Treatment with COVID-19 Incidence. LID - 2020.05.08.20095893 [pii] LID - 10.1101/2020.05.08.20095893 [doi].

2) Mitchell F. Vitamin-D and COVID-19: do deficient risk a poorer outcome? (2213-8595 (Electronic)).

3) Karohl C, Su S Fau - Kumari M, Kumari M Fau - Tangpricha V, Tangpricha V Fau - Veledar E, Veledar E Fau - Vaccarino V, Vaccarino V Fau - Raggi P, et al. Heritability and seasonal variability of vitamin D concentrations in male twins. (1938-3207 (Electronic)).

Reviewer #2: In this paper authors investigate whether genetically predicted vitamin D levels are associated with Covid-19 susceptibility and severity using Mendelian randomisation approach. The rationale for the work is very well justified, and the work conducted is impressive. However, I have a few major comments that would need to be addressed before this study is published:

**We thank the reviewer for their insightful comments.**

1. Context relating to respiratory tract infections and vitamin D RCTs is misrepresented

Dozens of RCTs investigating the role of vitamin D in respiratory tract infections were conducted to date; majority show benefit (either statistically significant, or non-significant with OR suggestive of benefit). In particular see:

Martineau AR et al. Vitamin D supplementation to prevent acute respiratory tract infections: systematic review and meta-analysis of individual participant data. BMJ. 2017;356:i6583. Published 2017 Feb 15. doi:10.1136/bmj.i6583

In the Introduction, statement associated with Reference 14 (line 123) therefore misrepresents the context. Of note, cited paper found increased risk in treated arm for upper respiratory tract infections, but not for lower respiratory tract infections - later probably being more relevant in the context of Covid-19.

**We thank the reviewer for bringing this to our attention. While it would be difficult to provide a full critical appraisal of this individual patient meta-analysis and its constituent cohorts, it should be noted that its primary outcome was any respiratory tract infection diagnosis. As the reviewer points out, this is unlikely to be a meaningful outcome since it will be largely driven by benign upper respiratory tract infections. We also worry that this choice of outcome is very likely to be highly sensitive to how carefully each trial’s investigators were looking for upper respiratory tract infections, as well as local microbiological or clinical diagnostic modalities.**

**Moreover, the adjusted odds ratio of lower respiratory tract infection in this same meta-analysis was 0.96 (95% CI 0.83 to 1.10) and the adjusted all-cause mortality odds ratio was 1.39 (95% CI 0.85 to 2.27). Acknowledging that confidence intervals crossed the null, as outcomes become more clinically meaningful and diagnostically reliable, vitamin D supplementation in these randomized controlled trials was associated with very little benefit and even numerically worse outcome.**

**We have modified this section of the introduction, which now reads the following:**

***“In the field of infectious diseases, an individual patient data meta-analysis of randomized controlled trial of 25OHD supplementation[14] showed some benefit to prevent respiratory tract infections (OR 0.80, 95% CI: 0.69 to 0.93). However, this effect was driven by generally benign upper respiratory tract diseases, and was not observed in lower respiratory tract infections (OR: 0.96, 95% CI: 0.83 to 1.10) and even showed numerically worse all-cause mortality (OR: 1.39, 95% CI: 0.85 to 2.27).”***

Moreover, Castillo et al. published a RCT that high-dose vitamin D supplementation significantly reduced need for ICU (ie decreased risk of severe disease) - findings that are at odds with results reported here.

Castillo ME, Entrenas Costa LM, Vaquero Barrios JM, Alcala Dıaz JF, Miranda JL, Bouillon R, Quesada Gomez JM, E¨ ffect of Calcifediol Treatment and best Available Therapy versus best Available Therapy on Intensive Care Unit Admission and Mortality Among Patients Hospitalized for COVID-19: A Pilot Randomized Clinical study¨, Journal of Steroid Biochemistry and Molecular Biology (2020)

**We thank the reviewer for pointing out this paper. We were unaware of the Entrenas Castillo et al trial, which was published less than a week before we submitted our manuscript for review. While this trial raises multiple red flags about bias in reporting its results (e.g. no patient dropped out, no patient refused the intervention, varying follow-up time, etc.), its effect size is also unheard of in medicine apart from very rare medications like tyrosine kinase inhibitors in chronic lymphocytic leukemia. Moreover, it suffers from a very small sample size (especially in the control arm) and was open label. In cases where outcomes depend on treating physician decisions (like intensive care unit admission), an open label is highly prone to bias. The small number of deaths (two) compounded with the above problems make it very difficult to draw meaningful conclusions on the use of vitamin D in covid-19 from this study.**

We have added the following to the introduction:

***“For now, we are only aware of one RCT on vitamin D supplementation in Covid-19 [16], which showed less intensive care unit admissions in the treatment arm. However, the combination of its small size (n=75), varying follow-up time for mortality, and open label design put it at high risk of bias and is unlikely to be able to inform physicians and public health authorities on vitamin D supplementation.”***

2. Need more information on cohorts, and on control definition and risk of misclassification

Cohorts are referenced, but information given in the paper (or supplementary) is not enough to critically understand the work and implications, particularly in relation to cohorts used - descriptive analysis covering: age, gender, period covered (case/control assessment during which months of 2020?), countries included, ethnicity, comorbidities, BMI…).

I suggest expanding Table 1 to include this for all cohorts used.

Among cohorts used, are disease outcomes harmonised?

Misclassification (likelihood of false negatives in particular) is very likely and it would be good to report what it might be, for example using prevalence of disease in the cohort and what was in the population cohort was sampled from at that time. Eg in UKBiobank it is reasonable to expect a high proportion of [mild or asymptomatic] cases among controls given that testing was not widespread - which also means mild cases are "missing" amongst cases.

**We have updated the case-control counts (due to the new data release) and added information on each cohort’s country and patient ancestries. These can be found in Supplement 1. Unfortunately, some of this other requested information is not currently available publicly from the Covid-19 Host Genetics Initiative, as they would require patient level data and a large amount of legal work that would have prevented the initiative from providing any results in the first place (similar to how Zhang et al [PMID 32972995] from the Covid-19 Host Genetics Effort provided only limited patient level information).**

**Further, while the other specific epidemiological characteristics were not made available by the Covid-19 Host Genetics Initiative, the genotyping quality control, GWAS analysis protocols, and outcome phenotype definitions are openly available, and were followed by all cohorts in order ensure valid comparisons and warrant inclusion in the meta-analysis. We believe that a pandemic and the need for prompt results justifies this compromise.**

**We have added this information in the method section as follows:**

***For our study, we used the Covid-19 HGI fixed effect meta-analysis of GWAS from up to 22 cohorts, performed in up to 11 countries. Every participating cohort was asked to provide summary statistics from a GWAS on the above three outcomes, and including the following non-genetic covariates: age, sex, age*age, age*sex, 20 genetic principal components, as well as any locally relevant covariates at the discretion of participating studies (e.g. hospital, genotype panel, etc.). Cohorts were asked to follow common sample and variant quality control, and only performed analysis if they enrolled 100 cases or more. Analyses were done separately for each major ancestry group to further control for population stratification. For the purposes of our study, we used the meta-analysis results from European ancestry cohorts, except for the severe Covid-19 cohort, for which this meta-analysis was not available. Further details on the three phenotypes and participating cohorts are found in Table 1 and Supplement 1.***

**Moreover, all outcome phenotypes were harmonized between studies using the definitions already provided in our manuscript (which are adapted from the Covid-19 Host Genetics Initiative). While we recognize that the hospitalization phenotype may be ascertained differently in each cohort (as criteria for hospitalization vary in different jurisdictions), and that the susceptibility outcome may misclassify asymptomatic cases as controls (though only a small minority of the UK population and the UK Biobank was indeed infected with SARS-CoV-2), the severity phenotype is highly unlikely to vary significantly. Importantly, if there were large differences in outcome ascertainment between cohorts, we would expect poor replication of genetic associations between cohorts. However, this was not reported by the initiative, with each reported association becoming more significant with each round of meta-analysis, more participating cohorts, and larger sample sizes (e.g. the chromosome 3 locus).**

3. Meaning behind the reported findings

What does "one standard deviation on the logarithmic scale" of genetically increased 25OHD level mean - ie. what is the corresponding increase in nmol/L in circulating 25OHD concentration associated with this?

**This is a good question from the reviewer, as we need to make sure that a standard deviation in vitamin D is comparable to what would be observed with exogenous supplementation. Indeed, the 25OHD standard deviation in the UK Biobank is 21.1 of nmol/L. Given that the UK Biobank made up 90.5% of the individuals included in the vitamin D GWAS meta-analysis, this is likely a good approximation. As a comparison, when taking daily 25OHD supplement at a dose of 2000IU (more than usual practice) for 3 weeks, Żebrowska et al (PMID:32272973) found a rise of 13.5 nmol/L.**

**We have clarified this in the results section with the following:**

***“Of note, a one standard deviation increase in 25OHD serum levels translated to a rise of 21.1 nmol/L of 25OHD in the UK Biobank population, a rise that is comparable to what can be achieved with vitamin D supplementation, especially in short courses[40].”***

Given that vitD genetic proxy explains 1-4% of variance, it probably means that only a very small difference in circulating 25OHD (couple of nmol/L perhaps?) can be explained by the genetic proxy. Combined with the increasingly accepted notion that the relation between vitamin D and health outcomes follows a sigmoid (with threshold effect) rather than linear curve (Heaney R, 2013, Nutrition Reviews), is this finding strong enough to inform guidelines on vitamin d supplementation?

**The strength of mendelian randomization is that while the genetic instruments may only explain small percentages in exposure variance, the association is nevertheless translated on the exposure-outcome association scale. There is of course a limit to this, which is that when genetic instruments explain too little of the variance in exposure, the results will be biased towards the null. However, we know from previous literature that these same variants can provide non-null results (e.g. in multiple sclerosis, for which MR was used to support vitamin D supplementation [Mokry et al, Plos Medicine 2015, PMID 26305103]). Hence, it is unlikely that we are faced with a weak-instrument bias (we also refer to our responses to reviewer 3 for more detail on this issue below).**

**As for the sigmoidal shape being important to vitamin D and health outcomes, we refer to our discussion on non-linearity of the exposure-outcome relationship in our response to reviewer 1 (point 5). We also note that while a sigmoid may be a more appropriate model, previous MR studies have nonetheless obtained results that were in line with RCTs (which have usually shown no effect of supplementation, regardless of any purported sigmoidal association).**

4. Interpretation of the findings

Looking at main results table (Table 2), it would also be true to say:

"When we used genetic instruments that are most likely to affect Covid-19 outcomes via their effect on 25OHD (after remoal of SNPs identified by Phenoscanner), a statistically significant reduction in Covid-19 susceptibility was noted among those with higher genetically predicted 25OHD (OR=0.38, 95%CI: 0.19-0.75). A suggestive decrease in risk was also noted when analysis only included genes in the vitamin D pathway (OR=0.61, 95%CI: 0.37-1.01)."

Of note, these results are reported incorrectly in the current version of the manuscript (line 278: "….we…found.. increased 25OHD levels on susceptibility …".) - in all analysis, DECREASED risk for susceptibility was found (clear in Figure 2).

**We have now updated the analyses using updated Covid-19 Host Genetics Initiative results. The results are consistently null. However, it should be noted that the reviewer is referring to the analysis with the smallest number of genetic instruments, and the most likely to give biased effect estimate given its lower of statistical power (MR Egger), while not accounting for multiple testing. Nevertheless, our updated report using the updated meta-analysis do not support their interpretation of our results.**

It would be a more balanced paper if these findings were brought forward and discussed in the paper also - particularly how to explain different direction of the effect for susceptibility and severity.

**These results do not apply to the updated analysis, which now give consistent lack of association between 25OHD level and Covid-19 outcomes (allowing for minor differences in analytical method and expected statistical variation from multiple testing).**

Reviewer #3: This is an interesting paper, but I'm afraid that the results are strongly affected by selection / collider bias. For me, the primary analysis is valid, but the secondary analyses are faulty. The reason is that the primary analysis is confirmed absence versus presence of COVID-19, whereas the secondary analyses are severe versus mild COVID - hence even controls are selected as those who have COVID-19. A better analysis would be severe COVID versus no COVID. As per Table 1 of "Contextualizing selection bias in Mendelian randomization: how bad is it likely to be?" by Gkatzionis and Burgess, when selection into the sample depends on the risk factor (which is clearly the case for the secondary analyses here, as COVID status of participants would associate with vitamin D levels), bias is in the opposite direction as the observational association. Hence the COVID-19 severity analyses provide associations in the harmful direction. Hence the paper is interest, but currently mainly as an exposition of the bias that can arise due to improper selection of controls.

**We appreciate Professor Burgess’ insightful comments on this issue. Interestingly, the conclusion from the Gkatzionis et al paper is that the effect of selection bias on mendelian randomization estimates is substantial mainly when the effect of the risk factor on selection is large. Here, the effect of 25OHD levels on Covid-19 susceptibility is small, and the effect of vitamin D on being selected in the hospitalization and severe disease phenotype is also small. Therefore, the bias in our results in the hospitalization and severe diseases phenotypes is also likely to be small. Accordingly, Table 1 of the referred paper shows that for odds ratios of the selection effect between 0.61 and 1.65, the type 1 error rate is still less than 6% (assuming a 5% alpha significance threshold).**

**Nevertheless, the risk of selection bias persists, and we agree that using Covid-19 negative controls may be wise. While the Covid-19 Host Genetics Initiative does not provide outcome phenotypes with only Covid-19 negative controls, more than 95% of controls in the hospitalized and severe disease phenotypes are from population-based cohorts, with Covid-19 positivity rates of less than 1% (UK Biobank, FinnGen, and Decode, see Supplement 1, Table 1) at the time of data release. This is therefore likely a very good approximation of the proposed phenotype. We previously reported results of these phenotypes in the supplements but have now made them our primary results. The previous primary results were removed from this updated manuscript, due to their higher risk of collider and selection bias.**

Overall, this is an interesting paper, but I would encourage the authors to only perform analyses where the comparison group is population-based controls. My personal preference is severe COVID versus control, as severe COVID is less susceptible to selection (see https://www.medrxiv.org/content/10.1101/2020.06.18.20134676v1.full.pdf). But there are other GWAS datasets that are available (see https://www.medrxiv.org/content/10.1101/2020.09.15.20165886v1.full.pdf - although we did also consider severe versus mild disease, so not fully following my own advice!).

**As described above, we agree with this suggestion, and have therefore used those case-control definitions for our updated analyses.**

I think a paper on this subject would be interesting, but the results and message would likely change substantially if the authors concentrated on the primary analysis (which suggests a null result), so will only provide broad comments at this point:

1. Abstract: "Genetically increased 25OHD levels by one standard deviation on the logarithmic scale had no clear effect on susceptibility but tended to increase the odds ratio..." - I'd prefer to see non-causal language in the results section of the manuscript. What you observe is that "Genetically higher 25OHD levels... associate with the risk of hospitalization". What you infer from this is that 25OHD is not a causal risk factor, but you do not show this directly - the causal aspect of the manuscript is an inference from the findings, not the finding itself. In other places too - for me, causal language is fine when describing the aims and interpretation of the work (although should be clear that causal interpretation is subject to untestable assumptions), but not when describing the results of analyses.

**We have now removed causal language and only speak of an association, as follows:**

***“Using inverse-weighted MR, genetically increased 25OHD levels by one standard deviation on the logarithmic scale had no clear association with Covid-19 susceptibility (OR = 0.97; 95% CI: 0.95, 1.10; P=0.613), hospitalization (OR = 1.11; 95% CI: 0.91, 1.35; P=0.299), and severe disease (OR = 0.93; 95% CI: 0.73, 1.17; P=0.531).”***

2. Did you account for correlation between genetic variants? Even if the variants were conditionally independent in their associations with 25OHD, they may well be correlated in their distributions.

**We have now removed 4 previously allowed variants from our analysis, due to having linkage disequilibrium of more than coefficient (R squared) of more than 5%. The methods section was rewritten as follows:**

***“From the list of conditionally independent variants provided, we further selected SNPs whose effect on 25OHD level was genome-wide significant (P<5x10^-8^), minor allele frequency was more than 1%, and with linkage disequilibrium coefficients (r^2^) of less than 5%.”***

3. Given the recommendation to focus on the results versus controls that suggested associations in the protective direction (albeit not statistically significant), you may want to think again about weak instrument bias and the preference for one-sample MR. I don't feel strongly here, but if the results change, then the argument that weak instrument bias is unimportant as the estimates are in the harmful direction no longer holds.

**We agree with professor Burgess on the risk of weak-instrument bias. However, even weaker vitamin D instruments have been able to provide positive results that contrasted with epidemiological associations in other diseases (most significantly multiple sclerosis [Mokry et al, Plos Medicine 2015, PMID 26305103]), and we do not suspect weak instrument bias plays a major role here. Nevertheless, a one-sample MR is a good suggestion. In fact, given that close to 350,000 individuals used as severe phenotype controls were also used in the vitamin D exposure GWAS, this analysis is halfway between a one-sample and a two-sample MR. Yet, while the three odds ratios from the inverse-variance weighted analyses were numerically in favor of higher 25OHD, the results were still largely null, including in the sensitivity analyses.**

**We have added the following to the limitations section:**

***“Second, our study may suffer from weak instrument bias, especially the sensitivity analyses restricted to smaller sets of genetic instruments. In two-sample MR, this bias would pull our estimates towards the null. Nonetheless, similar studies have been able to use MR to establish an association between 25OHD levels and other disease (most notably multiple sclerosis[26]), suggesting that these instruments are strong enough to find associations. Further, given the large percentage of shared individuals from the UK Biobank between the vitamin D exposure GWAS[27] and the severe Covid-19 phenotype, this analysis is close to a one-sample MR, which would show bias towards the observational studies effect. Given that this analysis also shows largely null effects, we do not suspect that weak instruments bias is a significant issue in our results.”***

4. A further limitation is that summary data MR cannot address nonlinearity. This is not a serious limitation for the main public health question, which relates to large-scale population interventions, but it may still be that vitamin D supplementation has a benefit for those who are vitamin D deficient.

**We agree with this comment, and have added it as a possible limitation. As professor Burgess correctly writes, this is unlikely to be a serious limitation, as even in RCTs on vitamin D supplementation that controlled for vitamin D deficiency, supplements rarely led to different outcomes.**

In summary, if this paper is re-written to focus on the primary analysis and other analyses that suggest a null effect, then it would be a valuable contribution to the literature.

**We thank professor Burgess for his help in improving our manuscript.**
