# Supplementary material for: Vitamin D and COVID-19 susceptibility and severity in the COVID-19 Host Genetics Initiative: A Mendelian randomization study
Source: PLoS Med. 2021 Jun 1;18(6):e1003605. doi: 10.1371/journal.pmed.1003605 (PMC8168855; doi:10.1371/journal.pmed.1003605)
Supplement: Attachment — Submitted filename: Rebuttal.docx [file pmed.1003605.s009.docx]

Requests from the editors:

Title: Please include some information on the study population if feasible.

**The title was modified as follows: *“Vitamin D and COVID-19 Susceptibility and Severity in the COVID-19 Host Genetics Initiative: a Mendelian Randomization Study”.***

Abstract: Please rename the “Introduction” section to “Background”

**This was done as suggested.**

Abstract: Please spell out Mendelian randomization for MR at first use.

**This was done as suggested.**

Abstract: Background: Line 72-73: Please revise to mitigate causal language: “Here, we used  two-sample MR to assess evidence supporting a causal effect of circulating 25OHD levels on Covid-19 susceptibility.” or similar.

**This was done as suggested.**

Abstract: Methods and Findings: Please provide some detail regarding this GWAS: “Genetic variants strongly associated with 25OHD levels in a 443,734-participant genomewide association study (GWAS) were used as instrumental variables.”

**We have added that the population used for that GWAS was all of European ancestry, similar to how we described the COVID-19 outcome GWASs later in that section: *“Genetic variants strongly associated with 25OHD levels in a genome-wide association study (GWAS) of 443,734 participants of European ancestry (including 401,460 from the UK Biobank) were used as instrumental variables.”***

Abstract: Methods and Findings Line 79-80: Please clarify this sentence- are these two cohorts of individuals with COVID-19, or is one intended to be without COVID-19.  Also, please clarify briefly the manner of how it was determined that the individuals had COVID-19.

**These sentences of the Methods and Findings section were modified as follows:**

***“******GWASs of COVID-19 susceptibility, COVID-19 hospitalization, and COVID-19 severe disease from the COVID-19 Host Genetics Initiative were used as outcome GWASs. These included up to 14,134 individuals with COVID-19, and 1,284,876 without COVID-19, from 11 countries. SARS-CoV-2 positivity was determined by laboratory testing or medical chart review. Population controls without COVID-19 were also included in the control groups for all outcomes, including hospitalisation and severe disease.”***

Abstract: Conclusion: We suggest adding an opening sentence such as: “In this 2 sample MR study, we did not observe evidence to support an association between 25OHD levels and COVID-19 susceptibility, severity, or hospitalization.” or similar.

**This was done as suggested.**

Author summary: Why was the study done?: We suggest deleting the first bullet point.

**This was done as suggested.**

Author summary (and throughout the text): Please consistently capitalize the Mendelian in Mendelian randomization.

**This was done as suggested.**

Methods: Line 220-221: Please qualify this statement with “to the best of our knowledge” or similar.

**This was added to the end of the sentence as suggested: *“To find genetic variants explaining 25OHD levels[27], we used a GWAS from our group, which is the largest published GWAS of 25OHD levels, to the best of our knowledge.”***

Methods: Line 237-243: Please clarify the description to indicate, for the hospitalization and severity cohort, whether the controls were individuals without severe Covid/hospitalized for Covid and did or did not have Covid-19.

**These sentences were re-written for clarity as follows:**

***“We used the COVID-19 HGI outcome definitions and GWAS summary statistics for COVID-19 susceptibility, hospitalization, and severe disease outcomes[31]. For all outcome, a COVID-19 infection was defined as a positive SARS-CoV-2 laboratory test (e.g. RNA RT-PCR or serology tests) or electronic health record evidence of SARS-CoV-2 infection (using International Classification of Diseases or physician notes). The susceptibility phenotype compared COVID-19 cases, with controls which were defined as any individual without a history of COVID-19. The hospitalized outcome compared cases defined as hospitalized patients with COVID-19, and controls as any individuals not experiencing hospitalization for COVID-19, which includes individuals without COVID-19.COVID-19. The severe disease outcome cases were defined as hospitalized individuals with COVID-19 who required respiratory support. Respiratory support was defined as intubation, CPAP, BiPAP, continuous external negative pressure, or high flow nasal cannula. Controls for the severe COVID-19 outcome were defined as individuals without severe COVID-19 (including those without COVID-19). The inclusion of COVID-19 negative participants as controls in each outcome decreases the possibility of collider bias[32] and allows for a better population level comparisons.”***

Methods: Please add the following statement, or similar, to the Methods: "This study is reported as per the Strengthening the Reporting of Observational Studies in Epidemiology (STROBE) guideline (S1 Checklist).”

**The suggested sentence was added at the end of the first paragraphs of the Methods.**

Results: Line 320: Please change “clear” to “statistically significant” or similar, and please revise the sentence to: “For a standard deviation increase in log-transformed 25OHD level, we observed no significant association with odds of susceptibility (OR = 0.97; 95% CI:0.85, 1.10; P = 0.61).”

**This was done as suggested.**

Results: Line 321-324: To address the comment of Reviewer 2, please clarify the relationship between 1SD increase in biomarker vs instrument.

**We have done so, and we kindly refer the editor to our response to the reviewer below for a more detailed explanation. As stated below we have changed the sentence to the following:**

***“Of note, in the UK Biobank, the distribution of 25OHD levels has a mean of 48.6 nmol/L and a standard deviation of 21.1 nmol/L. This standard deviation is comparable to what can be achieved with vitamin D supplementation, especially over short therapeutic courses[41].”***

Results: Line 329: The word “clear” is ambiguous, it could be removed or replaced.

**This was done as suggested.**

Results: Line 324-326: Please revise to “Similarly, we observed no significant difference in risk of hospitalization (OR =1.11; 95% CI: 0.91, 1.35; P = 0.30) or risk of severe disease (OR = 0.93; 95% CI: 0.73, 1.17; P = 0.53) associated with a standard deviation increase in log-transformed 25OHD level (Table 2 and Figure 1).”

**This was done as suggested.**

Results: Line 331: To avoid overly causal language we suggest referring to associations when reporting the results.

**This was done as suggested. We replaced the word “effect” with the word “association”.**

Results Line 338-341: Please revise to “Using IVW, each standard deviation increase in log-transformed 25OHD was again not associated with Covid-19 susceptibility (OR = 0.962; 95% CI: 0.83, 1.11;P = 0.594), hospitalization (OR = 1.07 [95% CI: 0.78, 1.47]; P = 0.668) and severe disease (OR = 0.869; 95% CI: 0.635, 1.19; P = 0.378).”

**This was done as suggested.**

Results: Line 347-350: Please revise to “In both cases, the association with each phenotype was mild compared to their effect on 25OHD level, as rs11723621 explained less than 0.03% of the variance in white blood cell counts, and rs6127099 explained less than 0.001% of the glomerular filtration rate variance[39,40].”

**This was done as suggested.**

Results: Line 360: Please change “hypothesis” to “hypotheses”

**This was done as suggested.**

Discussion: Line 364-366: We suggest revising slightly to avoid overly causal language: “In this large-scale MR study, we did not find evidence to support that genetically increased 25OHD levels are protective against Covid-19 susceptibility, hospitalization, or severity. This lack of evidence supporting a causal association was consistent across phenotypes, sensitivity analyses, and choice of genetic instruments.” or similar.

**This was done as suggested.**

Discussion: Line 371-374: Please include the citation for the study described here. Also we would suggest revising to remove the first instance of the word “small” as it is emphasized later in the statement, and also clarify the “flawed endpoint” to make your point more apparent: “Further, while a small randomized trial showed benefit of vitamin D supplementation, this trial used a flawed endpoint and a small sample size, and it is therefore unable to invalidate our results.”

**We made the suggested changes to the sentence, but also incorporated more recent results from a larger RCT (with better endpoints) that showed no effect from vitamin D supplementation. The sentence now reads:**

***“Further, while a randomized trial[15] showed benefit of vitamin D supplementation using a an endpoint at risk of bias due to the unblinded intervention (admission to the critical care unit) and a small sample size (n=75), a larger randomized trial[41] of 240 patients showed no effect of a single high dose of vitamin D3 on mortality, length of stay, or risk of mechanical ventilation. These findings from the larger randomized trial are thus concordant with our MR results.”***

**Similarly, we changed the sentences in the introduction that start with “For now, we are aware of” to the following:**

***“At present, we are aware of two RCTs testing the role of vitamin D supplementation on COVID-19 outcomes, both using high-dose vitamin D given at time of hospital admission for COVID-19. The first [15] was a small trial (n=75) showing less intensive care unit admissions in the treatment arm. However, the follow-up time for mortality varied, and the open label design put it at high risk of bias. The second[16] was a larger study (n=240) using a double-blind design, and showed no association on mortality, risk of mechanical ventilation, and length of stay. Nevertheless, questions remain on the use of pre-illness vitamin D supplementation.”***

Discussion: Line 404: Please revise to “...for the purpose of vitamin D supplementation…”

**This was done as suggested.**

Discussion: Line 406: Please revise to “...as we only studied the effect of 25OHD and Covid-19 in individuals of European ancestry, it remains possible…”

**This was done as suggested.**

Checklist: We agree the utility of the STROBE for case-control studies is limited, and the STROBE-MR does not seem to have been finalized/published.  If the authors feel the STREGA extension on the STROBE items is most appropriate, please use that.

**We agree with the current use of the STROBE checklist, and there is likely a benefit to using a checklist that is better known to the general readership.**

Figure 1: Would it be possible to include a note in the legend that p values are given in Supporting Information table 4 (if I have that correct)?

**The reviewers are correct, the p-values were in Supplement 4 (now named supplement 5). This following was added to the caption: *“Full results including odds ratios, confidence intervals, and p-values are available in Supplement 5.”***

Page 11: The sections Data Availability, Funding Source, Competing Interest, and Transparency statement can be removed from the main text of the manuscript.  Please ensure that all information is entered in the appropriate place in the manuscript submission forms (Financial Disclosure, Competing Interests, Data Availability)

**This was done as suggested.**

Comments from the reviewers:

Reviewer #1: Authors have answered my comments. I still have a minor suggestion, as follows,

The main finding that genetically increased 25OHD levels did not protect against COVID-19 susceptibility, hospitalization, or severity, indicates that endogenous 25OHD levels are not causallly associated with COVID-19 susceptibility, hospitalization, or severity, but not completely equal to the main conclusion. I suggest the authors to make the conculusion more carefully.

**We thank the reviewer for their help in improving the manuscript. We agree with their last suggestion and have now adjusted our conclusion to provide a more cautious interpretation of the relationship between genetically increased 25OHD and vitamin D supplementation. For example, the following sentences were added to the first paragraph of the discussion:**

***“While our study assessed the association between genetically determined levels of 25OHD and COVID-19, this can still inform us on the role of vitamin D supplementation. Specifically, in contrast to observational studies, our findings do not support an association between higher 25OHD level and better COVID-19 outcome, and therefore do not support the use of vitamin D supplementation to prevent COVID-19 outcomes.”***

**The last paragraph as also been modified as follows:**

***“In conclusion, using a method that has consistently replicated RCT results from vitamin D supplementation studies in large sample sizes, we find no evidence to support a protective role for higher 25OHD on COVID-19 outcomes. Specifically, vitamin D supplementation as a public health measure to improve COVID-19 outcomes is not supported by this Mendelian randomization study.”***

Reviewer #2:

Findings from this study have much improved after re-analysis with the most up-to-date dataset available. Process is streamlined, results clearer and presentation improved.

**We thank the reviewer for their help in improving our manuscript.**

It is unfortunate however that other sections of the paper have deteriorated since the original submission and paper needs substantial editing for coherence, typos, accuracy and most notably context and interpretation of findings.

For example:
- abstract line 80 should presumably state "…1,284,876 WITHOUT Covid-19…"
- authors inconsistently use "vitamin D level" when they mean "genetically predicted 25OHD level" (eg Author Summary)

**These were fixed as suggested.**

Major concerns at this stage are as follows:

1. In terms of methodology, the key issue with genetic instruments in this case is not that the instrument is weak, but that it's association with outcome is time-varying.

**Please see our next response.**

Genetic instruments explain large fraction of the variance in winter, but virtually none in the summer. This issue has been flagged in various MR contexts eg.
<https://www.ncbi.nlm.nih.gov/pmc/articles/PMC5123677/>
<https://academic.oup.com/aje/article/188/1/231/5098387>

**We note that the reviewer points out two studies which describe the importance of considering time-varying exposures in MR studies. We worry that the reviewer overstates the lack of effect of genetic instruments on 25OHD level in the summer. For example, in a recent study done in a Finnish population (where the effect of season on 25OHD is likely to be important due to large seasonal variation in sun exposure), a genetic risk score based on the number of genetic risk alleles associated with 25OHD was clearly able to discriminate between participants with high and low 25OHD levels, regardless of the season (figure 3 in this paper:** [**doi.org/10.1093/jn/nxaa391**](https://doi.org/10.1093/jn/nxaa391)**).**

**Most importantly, the results of 25OHD MR studies have been widely replicated by large-scale RCTs of vitamin D supplementation. Specifically, large-scale vitamin D supplementation RCTs to prevent cancer and cardiovascular outcomes have shown null effects, with 95% confidence intervals around the null. These null effects were anticipated by MR studies on these same outcomes, published prior to the RCTs. Further the large-scale D2d trial which tested the effect of vitamin D supplementation of on type 2 diabetes also showed a null effect (doi.org/10.1056/NEJMoa1900906). These results were also anticipated by large MR studies. MR studies of vitamin D on falls and BMD have also been concordant with RCTs on these outcomes (doi:10.1001/jama.2010.594 and doi:10.1136/bmj.k3225).**

**Thus, the weight of available evidence suggests that MR studies of vitamin D tend to converge with large-scale RCT results.**

In the discussion section, authors need to explain how the time-varying relationship between the instrument and the biomarker might have affected their null-findings, given that this has not been incorporated in the analysis.

**Please see our previous response. We have now added a sentence on the results from the Finnish cohort to further support our Mendelian randomization, while still acknowledging that the cyclical pattern is not entirely captured by a two-sample Mendelian randomization design:**

***“Third, given that vitamin D levels are affected by season (with higher levels after sunlight exposure), even if our SNP-instruments were obtained from a GWAS that controlled for season of blood draw, there is still a chance for effect attenuation by averaging the effect of 25OHD levels on COVID-19 over all seasons. Nevertheless, a recent study in a Finnish cohort (where sun exposure greatly varies with seasons) showed that genetic determinants of 25OHD level were able to discriminate between individuals with predisposition to varying levels of 25OHD, regardless of the season[46]. Therefore, while the cyclical nature of 25OHD level is not completely modelled by MR, the size of this bias is likely small.”***

2. The level of biased presentation remains highly concerning.

For example, the original submission cited selected finding from one RCT on respiratory tract infections, that showed an increased risk with vitamin D. After the individual-patient data meta-analysis of ~30 RCTs that published in BMJ was brought to authors' attention (<https://www.bmj.com/content/356/bmj.i6583>) - a study that showed stat sign beneficial effect of vitD overall (adjusted odds ratio 0.81, 0.72 to 0.91), and statistically significant effect among vitD deficient with substantial effect size (adjusted odds ratio 0.30, 0.17 to 0.53), authors choose to cite the statistically insignificant but "numerically worse all-cause mortality" outcome form this study (introduction line 160).

**Perhaps this was not seen by Reviewer #2, but the potentially beneficial effect of supplementation on overall respiratory tract infections was already reported in our introduction *(“In the field of infectious diseases, an individual patient data meta-analysis of randomized controlled trial of 25OHD supplementation[13] showed some benefit to prevent respiratory tract infections (OR 0.80, 95% CI: 0.69 to 0.93).”*). Our choice of then reporting on lower respiratory tract infections and mortality was due to the fact that the positive outcome was driven by largely benign upper respiratory tract infections, which are of lesser clinical consequence. This was a concern that was shared by the reviewer in their previous review, and with which we agreed.**

**We also note that the analysis restricted to individuals with vitamin D deficiency was only performed for the primary outcome, and a similar analysis for the lower respiratory tract or mortality outcomes was not performed (or was not shown). This is compounded by the fact that performing a secondary post-hoc analysis restricted to vitamin D deficient individuals is likely to cause selection bias, even when done in the context of an RCT, as vitamin D deficient individuals enrolled in the RCT are likely different from those that were not.**

In the refs to support the agreement between non-significant MR and RCTs, authors provide refs (Introduction line 179), but fail to mention that while statistically not significant, "numerically beneficial" effect of vit D (or OR at 1) was found in majority of primary and secondary analysis reported in these.

**We do not believe a full critical appraisal of primary and secondary outcome in each referenced RCT is of benefit to the introduction in the paper. However, we agree that there is a difference between not showing a clinical benefit, and not showing a statistically significant benefit. Hence, we have modified our previous sentence to clarify that previous RCTs may not have failed to rule out clinical benefit, and only failed to show a statistically significant effect, as follows:**

***“However, across endocrinology, respirology, cardiology, and other specialties, these trials have most often not demonstrated statistically significant benefits[9–11].”***

Authors do not mention vitD MR studies that found a stat sign beneficial effect of genetically predicted vitD level, eg <https://www.ncbi.nlm.nih.gov/pmc/articles/PMC4582411/>

**We are aware of multiple studies showing the potential benefits of vitamin D in disease certain disease outcomes. In fact, our group published the first MR study of the beneficial effect of 25OHD on risk of multiple sclerosis, which is referenced in the manuscript. In the previous version of the manuscript, this was only mentioned in the discussion, we have now added a reference to it in the introduction as follows:**

***“In the case of vitamin D, MR has been able to provide causal effect estimates consistently in line with those obtained from RCTs[9,20–24], or support the use of vitamin D supplementation in preventing diseases in at risk individuals (most notably multiple sclerosis disease[25]). Hence, MR may support investments in 25OHD supplementation trials in COVID-19, if a benefit was shown.”***

Authors might consider citing observational vitD study in UK Biobank
<https://www.ncbi.nlm.nih.gov/pmc/articles/PMC7204679/>

**We note that a main objective of Mendelian randomization is to move away from observational studies in vitamin D, since they are prone to subject to potential bias by confounding. Specifically, many observational studies of 25OHD level have shown protective associations with disease outcomes, which have not been validated in RCTs.COVID-19**

3. How did authors calculate that 1 SD in genetically predicted 25OHD corresponds to 21.1 nmol/L?

**Please see our next response.**

Mean 25OHD in entire UK Biobank is 48.6 nmol/L and SD is 21.1 nmol/L. One SD in the instrument is not equal to 1 SD in the biomarker, particularly if instrument explains only ~4% of the variance.

**We had previously only noted that a one standard deviation increase 25OHD was equivalent to a 21.1 nmol/L increase in the UK Biobank (“Of note, a one standard deviation increase in 25OHD serum levels translated to a rise of 21.1 nmol/L of 25OHD in the UK Biobank population”). This is very different from saying that genetics explain a change of 21.1 nmol/L (see more about the effect of genetics on 25OHD levels in our next response). The 21.1 nmol/L value is publicly available on the UK Biobank data showcase website (**<https://biobank.ndph.ox.ac.uk/showcase/field.cgi?id=30890>**). Again, we emphasize that the Mendelian randomization result provides a measure of association on the exposure (25OHD) to outcome (COVID-19) scale, and not on the genetics-to-exposure scale. Therefore, as long as the genetic instruments are not weak, Mendelian randomization is robust to the issues brought up by the reviewer. Nevertheless, we see that our previous sentence may have caused some confusion, and we have adjusted it as follows:**

***“Of note, in the UK Biobank, the distribution of 25OHD levels has a mean of 48.6 nmol/L and a standard deviation of 21.1 nmol/L. This standard deviation is comparable to what can be achieved with vitamin D supplementation, especially in short courses[41].”***

Given the authors' previous study (ref 27), it should be easy and it would be informative to use actual UK Biobank data to show the correlation between instruments (genetically predicted 25OHD) and actual 25OHD level. For example, creating quintiles of genetically predicted vitD level and giving mean and SD of measured 25OHD for each quintile in genetically predicted 25OHD.

**Given the polygenic nature of 25OHD levels, the most appropriate way to do this would be through a polygenic risk score, which requires dedicated analyses and validation. This is outside the scope of our current work and has already been published on multiple occasions using many genetically diverse cohorts (e.g. UK biobank with validation in a separate Italian cohort: doi.org/10.1212/WNL.0000000000007136, Finnish cohort: doi.org/10.1093/jn/nxaa391, Chinese cohort: doi.org/10.1186/s12937-019-0494-7). The results are all concordant, in that genetic determinants of 25OHD are found in the same genetic loci, and correlate with measured 25OHD. Interestingly, in the Finnish cohort (a population in which season is likely to greatly affect 25OHD level), the genetic risk score was able to explain differences in 25OHD between of 10 to 20 nmol/L, regardless of the season. Therefore, we do not think that repeating such an analysis would add to the already available body of work on genetic determinants of 25OHD.**

4. Interpretation of findings is not in line with results.

Eg last line of Abstract/results states that results do not apply to vitD deficient individuals. Appropriate conclusion should therefore be: "Our results do not support that individuals who are vitamin D sufficient be advised to take vitD supplements."

**We have changed the previous sentence to the following, which we feel strikes a good balance between broad recommendations about supplementation, and the likely minor population level bias caused by vitamin D deficiency:**

***“Hence, vitamin D supplementation as a mean of protecting against worse COVID-19 outcomes is not supported by genetic evidence.”***

In terms of guiding future research, a more constructive recommendation should be building on well-known issues with vitamin D RCTs - for example recruiting vitamin D deficient individuals in the trial (it is established that high proportion of vitD sufficient individuals in the trial limits the study from detecting the beneficial effect, as no futher benefit can be expected among those who are vitD sufficient at baseline). Or recommendation for future MR studies in this area - eg using a design that allows for a time-varying relationship that exists between the instrument and biomarker.

**Given the good correlation between MR and RCTs in vitamin D studies, we believe that recommending that RCTs be performed in vitamin D deficient individuals is a much stronger statement than our results allow us to make. Concerning the time-varying effect, we refer to our previous response above COVID-19.**

In terms of using MR to prioritise drugs for testing in RCT, there are two things to consider:
1) many potential medicines do not have a naturally occurring biomarker and cannot be interrogated in MR setting so it's hard to rank them against those that do

**We agree, though this does not affect our results or their interpretations.**

2) vit D is suitable for co-administration and hence factorial trial design

**We agree that vitamin D is suitable for co-administration with many drugs. However, this is not the topic of our paper.**

Reviewer #3: The authors have responded appropriately to the reviewer comments, and the paper is much improved. Couple of very minor comments (in no order of importance!):

**We thank the reviewer for their helpful comments in improving our manuscript.**

1. Line 336: "Second, we restricted out analysis" should be "Second, we restricted our analysis".

**This was fixed as suggested.**

2. Line 188: "However, this assumption still provides valid results" - For me, this could be clearer. The property is this: causal estimation in Mendelian randomization assumes a linear causal relationship between the exposure and outcome. But if the relationship is truly non-linear, then the estimate is still a valid test of the causal null hypothesis, and the estimate represents the population-averaged effect of a shift in the distribution of the exposure.

**We thank the reviewer for this insightful clarification, and the sentence now reads:**

***“However, this assumption still provides valid test of the null hypothesis when studying population-level effects[27], as MR then measures the population-averaged effect on the outcome of a shift in the distribution of the exposure.”***

3. "rejected the null hypothesis of lack of heterogeneity" - Would "homogeneity" be clearer than "lack of heterogeneity"? Also, it's not clear what is being referenced here - heterogeneity/homogeneity in what? I presume from context in the variant-specific causal estimates, but it's not clear from the text ("heterogeneity in the Covid-19 hospitalization phenotype" seems to suggest something else).

**We thank the reviewer for this suggestion and have clarified this sentence as follows:**

***“We nonetheless observe that for at least one of the three analyses, we would have rejected the null hypothesis of homogeneous genetic effects in the COVID-19 hospitalization phenotype.”***

4. Line 281 - "that effects Covid-19 outcomes" -> "that affects Covid-19 outcomes"

**This was fixed as suggested.**

5. Acknowledgement: the Supplements here are incorrectly numbered.

**This was fixed as suggested.**
