# Supplementary material for: Vitamin D and COVID-19 susceptibility and severity in the COVID-19 Host Genetics Initiative: A Mendelian randomization study
Source: PLoS Med. 2021 Jun 1;18(6):e1003605. doi: 10.1371/journal.pmed.1003605 (PMC8168855; doi:10.1371/journal.pmed.1003605)
Supplement: Attachment — Submitted filename: Rebuttal.docx [file pmed.1003605.s010.docx]

Requests from Editors:

1. Title: Please capitalize the first word in the subtitle, and use sentence case throughout: “Vitamin D and COVID-19 susceptibility and severity in the COVID-19 Host Genetics Initiative: A Mendelian randomization study"

**This was done as requested.**

2. Abstract: Line 89: Please revise "clear" to "significant" or "statistically significant" in this sentence.

**We replaced “clear” with “significant”, as requested.**

3. Abstract: Conclusion: Line 100: Please change “mean” to “means” in this sentence.

**This was done as suggested.**

4. Author Summary: What do these findings mean?  We suggest revising the first bullet point, to focus more on the findings/advance of your particular study- “Vitamin D is a highly confounded variable, and traditional observational studies are at high risk of biased estimates.”

**We still felt that emphasizing the difference between Mendelian randomization and traditional observational studies was important, especially for the uninitiated readers. However, we agree that the previous bullet point should be more clearly focused on our findings. Therefore, we combined the first two bullet points in that section, which now read as follows:**

***“Using Mendelian randomization to reduce confounding that has traditionally biased vitamin D observational studies, we did not find evidence that vitamin D supplementation in the general population would improve COVID-19 outcomes.”***

5. Author Summary: What do these findings mean? We suggest revising to: “Taken together with literature supporting concordance between MR studies and RCTs investigating vitamin D effectiveness, these findings suggest that other therapeutic and preventative avenues should be prioritized for COVID-19 trials.” or similar.

**This was done as suggested. As specified in the Author summary instructions, we did not use acronyms.**

6. Throughout: Please include a space between the preceding word and bracket of the in-text citation.

**This was done as suggested.**

7. Introduction: Line 212: Please revise the final sentence: “...to test the relationship between genetically increased 25OHD level on COVID-19 susceptibility and severity.” or similar, to avoid causal implications.

**As suggested, this sentence now reads: *“[…] an MR study to test the relationship between increased 25OHD level and COVID-19 susceptibility and severity.”***

8. Methods: Line 289: We suggest “undertook multiple analyses” rather than “undertook extensive analysis” here.

**This was done as suggested.**

9. Discussion: Lines 392-395: Please clarify this sentence: “These findings highlight the confounded association between vitamin D and COVID-19 due to factors such as older age, institutionalization, or medical comorbidities, that are all linked to lower vitamin D levels and cannot be controlled for even when using advanced statistical adjustments.”  Perhaps: “Differences between our findings and those reported in observational studies [references] may reflect the fact that associations between vitamin D and COVID-19 may be confounded due to factors difficult to control for even with advanced statistical adjustments, such as older age, institutionalizaiton or medical comorbidities linked to lower vitamin D levels.” or similar.

**This was done as suggested, with appropriate reference.**

10. Reference List:  Please double check the formatting of all references, and please use the "Vancouver" style for reference formatting, and see our website for other reference guidelines <https://journals.plos.org/plosmedicine/s/submission-guidelines#loc-references>. 
    For example, please indicate that # 16 is a preprint.  For # 25, PLOS Med should be PLoS Med.

**All references were reviewed, and the PLoS Medicine format was used (using the downloadable style file). Note that the previously cited pre-print (#16) is now published in JAMA, and the reference was adjusted accordingly. Similarly, reference 25 now reads PLoS Med, as requested.**

11. Table 1: Please define all abbreviations in the legend, including GWAS, UKB, CPAP, BiPAP.

**This was done as suggested. We have also added a definition for 25OHD.**

12. Checklist: Thank you for including the STROBE checklist.  Please make your responses more distinct from the checklist items, if possible (italics for your notes, or a separate column on the right).

**We have reviewed and update all sections of the checklist and put our responses in italics.**
